# MLST typing of *Treponema pallidum* subsp. *pallidum* in the Czech Republic during 2004-2017: Clinical isolates belonged to 25 allelic profiles and harbored 8 novel allelic variants

**DOI:** 10.1371/journal.pone.0217611

**Published:** 2019-05-31

**Authors:** Eliška Vrbová, Linda Grillová, Lenka Mikalová, Petra Pospíšilová, Radim Strnadel, Eliška Dastychová, Martina Kojanová, Miluše Kreidlová, Daniela Vaňousová, Filip Rob, Přemysl Procházka, Alena Krchňáková, Vladimír Vašků, Vladana Woznicová, Monika Dvořáková Heroldová, Ivana Kuklová, Hana Zákoucká, David Šmajs

**Affiliations:** 1 Department of Biology, Faculty of Medicine, Masaryk University, Brno, Czech Republic; 2 Department of Dermatovenerology, Faculty Hospital Brno, Brno, Czech Republic; 3 Department of Medical Microbiology, Faculty of Medicine, St. Anne's Hospital and Masaryk University, Brno, Czech Republic; 4 Department of Dermatology, 1st Faculty of Medicine, Charles University in Prague, Prague, Czech Republic; 5 Institute of Medical Biochemistry and Laboratory Diagnostics of the General University Hospital, The First Faculty of Medicine of Charles University in Prague, Prague, Czech Republic; 6 Department of Dermatovenerology, 2nd Faculty of Medicine, Charles University in Prague, Prague, Czech Republic; 7 Outpatient STI Clinic Medicentrum, Prague, Czech Republic; 8 National Reference Laboratory for Diagnostics of the Syphilis, National Institute for Public Health, Prague, Czech Republic; Universita degli Studi di Bologna Scuola di Medicina e Chirurgia, ITALY

## Abstract

A recently introduced Multilocus Sequence Typing scheme for *Treponema pallidum* subsp. *pallidum* was applied to clinical samples collected from 2004 to 2017 from the two largest cities (Prague and Brno) in the Czech Republic. Altogether, a total of 675 samples were tested in this study and 281 of them were found PCR-positive for treponemal DNA and typeable. Most of the typed samples (n = 281) were swabs from primary or secondary syphilis lesions (n = 231), and only a minority were whole blood or tissue samples (n = 50). Swab samples from patients with rapid plasma regain (RPR) values of 1–1024 were more frequently PCR-positive (84.6%) compared to samples from patients with non-reactive RPR test (46.5%; p-value = 0.0001). Out of 281 typeable samples, 136 were fully-typed at all TP0136, TP0548, and TP0705 loci. Among the fully and partially typed samples, 25 different allelic profiles were identified. Altogether, eight novel allelic variants were found among fully (n = 5) and partially (n = 3) typed samples. The distribution of TPA allelic profiles identified in the Czech Republic from 2004 to 2017 revealed a dynamic character with allelic profiles disappearing and emerging over time. While the number of samples with the A2058G mutation was seen to increase (86.7% in 2016/2017), the number of samples harboring the A2059G mutation was found to have decreased over time (3.3% in 2016/2017). In addition, we found several allelic profile associations with macrolide resistance or susceptibility, the gender of patients, as well as patient residence.

## Introduction

*Treponema pallidum* subsp. *pallidum* (TPA) is an unusual bacterial pathogen [1] that causes syphilis, a chronic venereal disease in humans. Each year, there are about 5.6 million new cases worldwide [2–3]. In recent years, there have been approximately 700–800 cases of syphilis per year in the Czech Republic, a country having about 10.5 million citizens (data provided by the Institute of Health Information and Statistics of the Czech Republic). Most of the cases were among men having sex with men (MSM), promiscuous individuals, or sex-workers [4].

While a syphilis diagnosis is mainly based on anamnestic data, clinical findings, and results of serological tests, PCR detection and molecular typing can also be used to diagnose syphilis, especially in the cases with negative serology [5]. Moreover, PCR and molecular typing have proved to be useful in cases where the infection was caused by *T*. *pallidum* subsp. *endemicum* [6–10].

In the last two decades, molecular typing of TPA isolates has mapped several thousand clinical isolates from different countries all over the world. During that time, the original typing technique [11] was continually improved for better resolution [12–13]. Sequencing-based molecular typing (SBMT) was introduced in 2006 [14] and was recently enhanced [15]. The recently reported treponemal MLST typing system analyses the TP0136, TP0548, and TP0705 loci, and has shown a resolution power of about 30% of whole genome sequences [15]. While MLST typing is a simple method revealing a high portion of genetic variability, whole genome sequencing is a more complicated method where just a subset of samples is usually characterized. In addition, 23S rDNA can be used to find mutations that cause macrolide-resistance, however, this analysis is not part of MLST. MLST typing has already been used to examine several hundred clinical samples from Switzerland [15], France [15, 16], and Cuba [17]. Moreover, a public treponemal MLST database for storage and analyses of typing data has been established [18].

In this communication, we performed molecular typing of TPA, using a newly introduced MLST typing system, on samples collected in the Czech Republic from 2004–2017. Some of the samples had been previously typed with the SBMT typing scheme [4, 19].

## Materials and methods

### Clinical material

Samples were collected during years 2004–2017 from two clinical departments in Brno (the Department of Dermatovenereology, St. Anne´s Faculty Hospital and the Department of Medical Microbiology, Faculty of Medicine, St. Anne´s Hospital and Masaryk University) and from two clinical departments in Prague (the Department of Dermatovenereology, 1st Faculty of Medicine, Charles University and the National Reference Laboratory for Diagnostics of Syphilis, National Institute for Public Health). Clinical data included patient age, gender, type of clinical material, results of serology, primary diagnosis, sexual orientation, and HIV status. Serological tests included *T*. *pallidum* particle agglutination (TPPA) or *T*. *pallidum* hemagglutination (TPHA) tests, the rapid plasma regain (RPR) test, and enzyme-linked immunosorbent assay (ELISA) or Western blot for IgM and IgG depending on the source hospital. Serological tests were provided by OMEGA Diagnostics (Reinbek, Germany), TEST-LINE (Brno, Czech Republic), and MARDX (Carlsbad, CA, USA). In total, 675 samples were examined by PCR. Patient characteristics for those with typeable samples (i.e., samples positive for at least one of typing loci) are given in Table 1.

**Table 1:** Clinical characteristics of patients with typed TPA samples.

Clinical characteristics of patients	Patients (n = 269)
Mean age (men/women)	44.2 (0–71)/24.9 (0–38)
Sex, n (%)	M 244 (90.71); W 25 (9.29)
Serology^a^	
TPPA/TPHA (%)	242 P (89.96); 7 N (2.6); 20 n.d. (7.43)
RPR	216 P (80.3); 47 N (17.47); 6 n.d. (2.23)
≤ 1:16	118
≥ 1:32	91
Positive without titer value	7
**Diagnosis**	
Primary syphilis stage	165
Secondary syphilis stage	36
Congenital syphilis	2
Undetermined syphilis stage	66
**Material**	**Samples (n = 281)**^**b**^
No. of swabs	231 (82.2%)
No. of whole blood samples	47 (16.7%)
No. of tissue samples	3 (1.1%)

P, positive; N, negative; n.d, not determined; M, men; W, women

^a^Serology is presented for the most frequently used tests.

^b^There were no differences found in multiple samples collected from one patient.

### Isolation of DNA

DNA was isolated as described previously [4] using QIAamp DNA Blood Mini Kits and a DNeasy Blood & Tissue Kits (Qiagen, Hilden, Germany).

### PCR amplification

Typing loci and the 23S rDNA locus were amplified as described previously [4, 15, 16, 20]. The PCR mixture (final volume of 25 μl) contained in the first step 16.3 μl of water, 2 μl of a 2.5 mM deoxynucleotide triphosphate (dNTP) mixture, 5 μl of 5x PS GXL buffer, 0.095 μl of each primer (100 pmol/μl), 0.5 μl of PrimeSTAR GXL polymerase (Takara Bio Europe, France), and 1 μl of isolated DNA. When PCR results were negative, the amount of isolated DNA per sample was increased: 11.5 μl of water, 2.5 μl of ThermoPol Reaction buffer, 0.5 μl of a 10 mM dNTP mixture, 0.25 μl of each primer (100 pmol/μl), 0.1 μl Taq polymerase (5,000 U/ml; New England BioLabs, Ipswich, MA) and 10 μl of DNA. In the first step, using 1 μl of DNA and GXL polymerase, PCR amplification was performed under the following condition: 94°C (1 min); 98°C (10 s), 68°C (15 s; −1.0°C per cycle), 68°C (1 min and 45 s) for 8 cycles; 98°C (10 s), 61°C (15 s), 68°C (1 min and 45 s) for 35 cycles; and 68°C (7 min). When using 10 μl of DNA and *Taq* polymerase, these following conditions were used: 94°C (1 min); 94°C (20 s), 55°C (20 s; −1.0°C per cycle), 72°C (1 min and 45 s) for 8 cycles; 94°C (20 s), 48°C (20 s), 72°C (1 min 45 s) for 35 cycles); and 72°C (7 min). The mixture for the second step was the same for both versions of the first step and the final volume (25 μl) for one reaction contained: 20.5 μl of water, 2.5 μl of ThermoPol Reaction buffer, 0.5 μl of a 10 mM dNTP mixture, 0.25 μl of each primer (100 pmol/μl), 0.1 μl *Taq* polymerase (5,000 U/ml; New England BioLabs, Ipswich, MA) and 1 μl of PCR product from the first step. PCR were performed under following conditions: 94°C (1 min); 94°C (30 s), 48°C (30 s), 72°C (1 min and 15 s) for 40 cycles; and 72°C (7 min). DNA of TPA strain Nichols (5 pg/μl) was used as a positive control; distilled water was used as a negative control. A list of all primers used for nested PCR is shown in S1 Table [15]. PCR products were purified using QIAquick PCR Purification Kits (Qiagen, Hilden, Germany) according to the manufacturer´s instruction.

### Sequencing and sequence analysis

We used Sanger sequencing performed at GATC Biotech AG (Constance, Germany; Eurofins Genomics Company). Analyses of the sequences were performed using Lasergene software (DNASTAR v.7.1.0; DNASTAR, Madison, WI, USA). Sequences were uploaded to the PubMLST database of *Treponema pallidum* subsp. *pallidum* [18] and allelic profiles were automatically assigned. Sequences of 23S rRNA genes encoding macrolide resistance or susceptibility were evaluated at positions corresponding to positions 2058 and 2059 in the 23S rRNA gene of *Escherichia coli* (accession no. V00331), where the A for G substitution has been shown to cause macrolide resistance [20–22]. These positions were carefully analysed to check for possible mix of wild-type and mutant sequences. Sequences were obtained by Sanger sequencing. Alleles encoding resistance were marked A2058G or A2059G depending on the site of substitution.

### Statistical methods

Correlations of characteristics of clinical samples with allelic profiles were performed using the two-sided Fisher´s exact test, and statistical significance was set at *p* < 0.05. Statistical analyses were performed using STATISTICA software v.12 (StatSoft, Tulsa, OK, USA).

### Ethics statement

This study was approved by the ethics committee of the Faculty of Medicine, Masaryk University (5G/2017). All patients provided written informed consent.

## Results

We examined 675 clinical samples collected from 2004–2017 from four hospitals in the two largest cities in the Czech Republic (2 hospitals in Brno and 2 hospitals in Prague). While all samples were tested against TP0705, TP0136, and TP0548 between 2014–2017, data for TP0136 and TP0548 came from clinical samples collected between 2004 and 2013 as part of previous studies [4, 19] and were retested in locus TP0705. We found 281 samples to be positive and typeable, i.e., at least one locus TP0136, TP0548, or TP0705, was amplified and sequenced. The majority of the typed samples were swabs from primary or secondary syphilis lesions (n = 231) and the rest were from whole blood samples (n = 47) and tissue samples (n = 3) taken *post mortem*. Most of the samples (86.48%) belonged to the SS14-like genetic group, while only 2.13% belonged to the Nichols-like genetic group. The remaining 32 samples (11.39%) were not classified as SS14-like or Nichols-like because of selective positivity for locus TP0705 which does not contain informative sites for discriminating between these two genetic groups. Clinical characteristics of patients are summarized in Table 1.

Swab samples from patients with RPR values from 1–1024 were more frequently PCR-positive (84.6%) compared to samples from patients with non-reactive RPR test (46.5%; p-value = 0.0001). No such difference was observed among whole blood samples, however, whole blood samples represented a minority of samples used in this study (16.7%; 47 out of 281).

**Fig 1:**
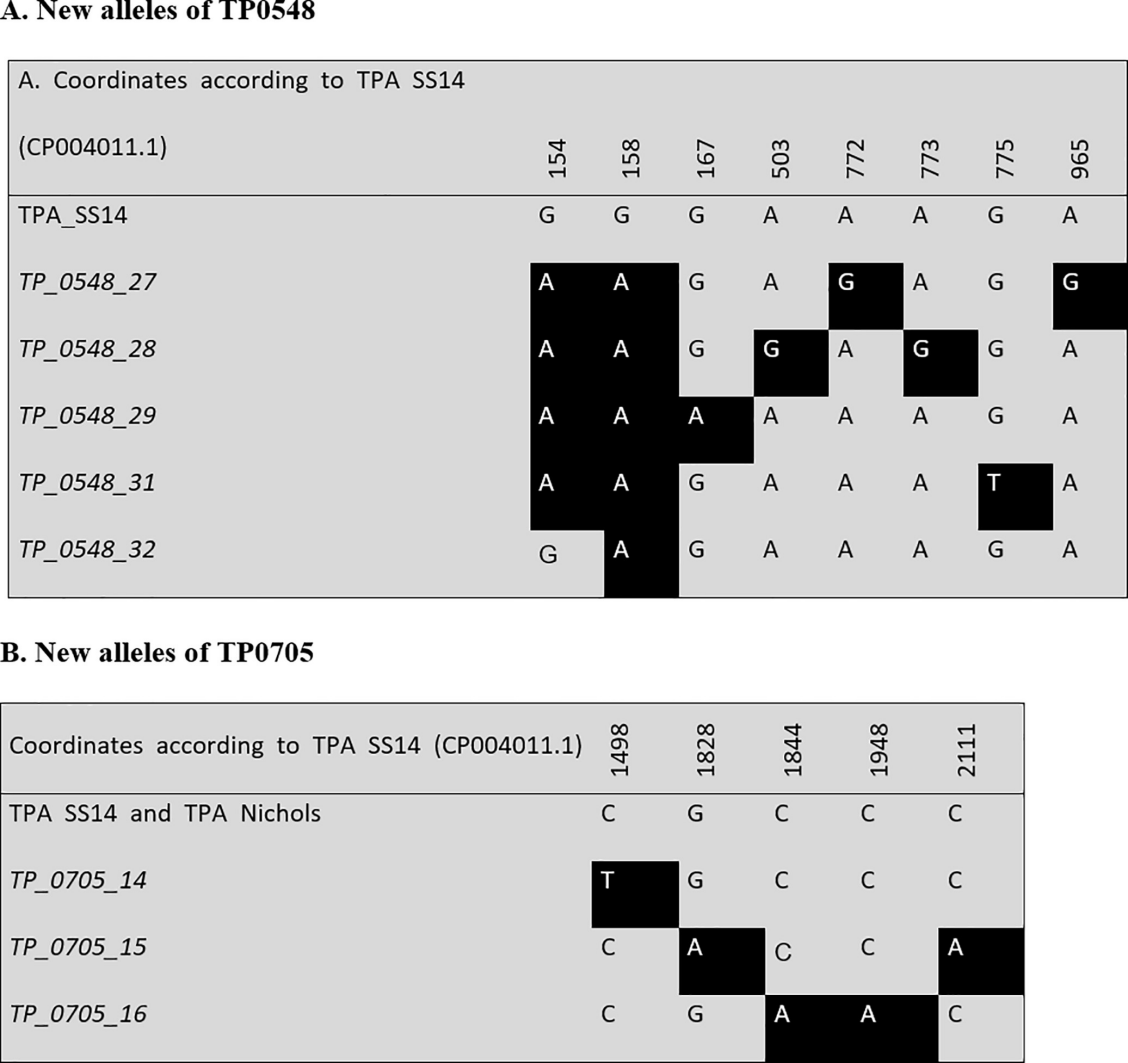
An alignment of the newly identified allelic variants. **A. New alleles of TP0548. B. New alleles of TP0705.** New alleles are in italics. A complete overview of allelic variants found in this study at the TP0136, TP0548, and TP0705 loci are shown in S1 Fig. No new alleles were found in the TP0136 locus.

Out of 281 typeable samples, 136 were fully-typed at the TP0136, TP0548, and TP0705 loci. Among the fully typed samples, 16 different allelic profiles were found, and the partially typed samples (n = 145) revealed 18 different allelic profiles (listed in S2 Table). Since nine allelic profiles were identified in both fully and partially typed samples, the total number of different allelic profiles identified in this study was 25. In fully-typed samples, five novel allelic variants were identified including four in TP0548 and one in TP0705. Novel allele variants (n = 3) were also identified among partially typed samples and included one in TP0548 and two in TP0705. Allelic profiles identified among fully-typed samples are summarized in Table 2, rest of identified variants are presented in S2 Table. The new allelic variants identified in this study are shown in Fig 1. Among both fully and partially typed samples, the highest amplification efficiency was found for locus 23S rDNA, which was not used for typing (positive in 233 samples), followed by TP0705 (typed in 224 samples), TP0548 (typed in 208 samples), and TP0136 (typed in 204 samples).

**Table 2:** Allelic profiles identified among fully-typed samples (n = 136).

Sequence type^1^	Allelic profile^2^	23S rDNA^3^ (no. of samples)	Genetic group [23].	No. of samples (%)
**1**	1.3.1.	S(1)/R8(44)/X(11)	SS14-like	56 (41.2)
**3**	1.1.8.	S(26)/R8(2)	SS14-like	28 (20.6)
**2**	1.1.1.	S(8)/R8(11)/X(1)	SS14-like	20 (14.7)
**25**	1.26.1.^4^	R8(10)/X(2)	SS14-like	12 (8.8)
**11**	1.1.3.	R9(5)	SS14-like	5 (3.7)
**44**	1.36.1.^4^	S(4)	SS14-like	4 (2.9)
**26**	9.7.3.	R8(1)/X(1)	Nichols-like	2 (1.5)
**48**	17.1.1.^4^	S(1)	SS14-like	1 (0.7)
**46**	1.31.1.^5^	R8(1)	SS14-like	1 (0.7)
**43**	1.28.1.^5^	R8(1)	SS14-like	1 (0.7)
**45**	4.1.1.	R8(1)	SS14-like	1 (0.7)
**41**	1.29.1.^5^	R8(1)	SS14-like	1 (0.7)
**50**	1.32.1.^5^	S(1)	SS14-like	1 (0.7)
**42**	1.1.16.^5^	R9(1)	SS14-like	1 (0.7)
**7**	1.4.1.	R8(1)	SS14-like	1 (0.7)
**49**	18.1.1.^4^	S(1)	SS14-like	1 (0.7)

^1^ According PubMLST database of *Treponema pallidum* subsp. *pallidum* [18].

^2^ Allelic profiles based on sequences of TP0136, TP0548, and TP0705 [15].

^3^ Locus encoding resistance to macrolide antibiotics: S = sensitive, R8 = A2058G mutation, R9 = A2059G mutation, X = undetermined. Both A2058G and A2059G mutations result in resistance to macrolide antibiotics.

^4^ Newly identified profiles, with known alleles from previous studies [4, 19]. Original description: 1.26. = SU5, 1.36. = SU7, 17.1. = U2S, 18.1. = U1S.

^5^ Newly identified allelic profiles, with novel alleles.

**Fig 2:**
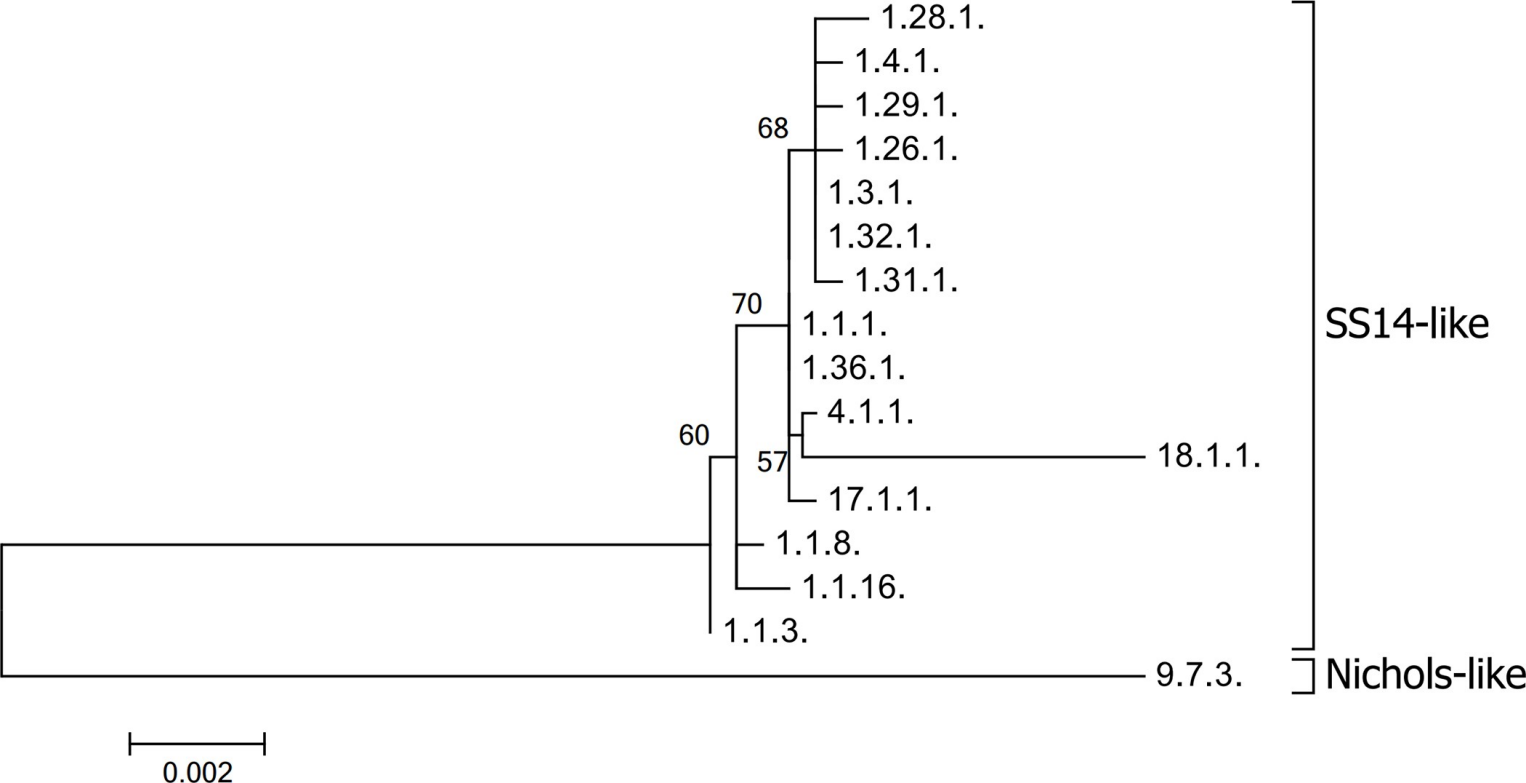
A phylogenetic tree of allelic profiles identified among fully typed samples. The scale shows the number of substitutions per site. Bootstrap values are shown next to branches. The length of concatenated sequences was 2593 bp and contained 95 variable positions. The tree was constructed in MEGA7 [24] using the Maximum Likelihood method [25] with the bootstrap test [26].

A phylogenetic analysis of allelic profiles found among fully typed samples is shown in Fig 2. The allelic profile 9.7.3. corresponds to the allelic profile of the Nichols-like strain, all other allelic profiles corresponded to the allelic profiles of the SS14-like strains. Except for the allelic profile 18.1.1., all identified allelic profiles of the SS14-like strains were highly related.

**Fig 3:**
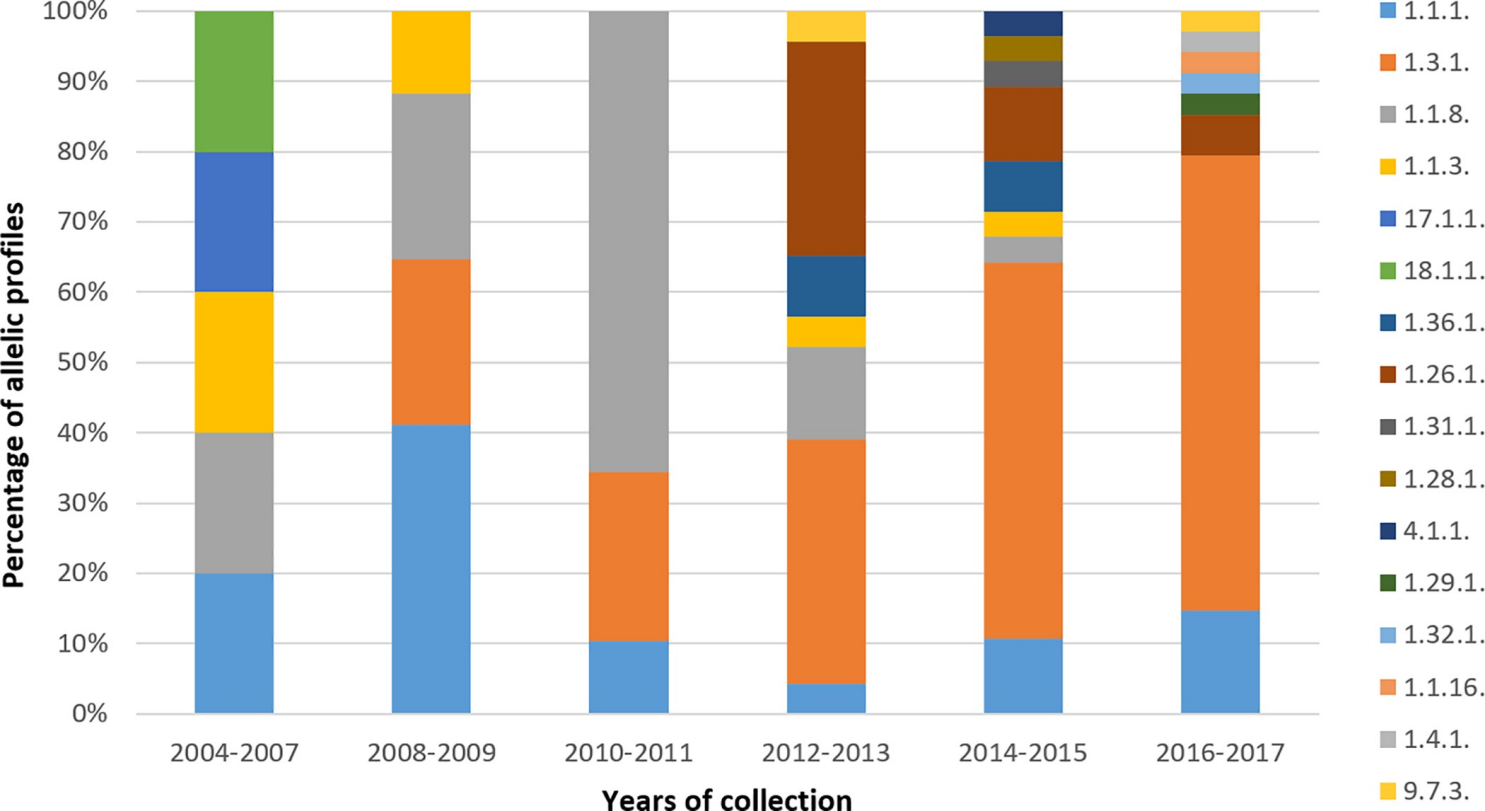
Distribution of TPA allelic profiles identified in the Czech Republic from 2004 to 2017.

The distribution of TPA allelic profiles identified in the Czech Republic from 2004 to 2017 is shown in Fig 3. Whereas allelic profile 1.1.8. (which was found to be associated with susceptibility to macrolides) reached its highest proportion in 2010 and 2011 and then disappeared in 2017, there were other allelic profiles that circulated during all tested years (e.g., 1.1.1.) or even increased (e.g., 1.3.1.). In 2012, a new allelic profile (1.26.1.) emerged and persisted until the end of the test period in 2017. Other allelic profiles seem to have random occurrence indicating the dynamic nature of circulating allelic profiles in particular populations. The most frequent was allelic profile 1.3.1. (41.2%) followed by allelic profiles 1.1.8. (20.6%), and 1.1.1. (14.7%). Moreover, as the number of samples increased (13 in 2004/2007, 24 in 2008/2009, 56 in 2010/2011, 63 in 2012/2013, 71 in 2014/2015 and 54 in 2016/2017), the number of allelic profiles also increased (11 in 2004/2007, 7 in 2008/2009, 8 in 2010/2011, 16 in 2012/2013, 17 in 2014/2015 and 13 in 2016/2017), suggesting that the genetic variability of TPA has not been fully described yet.

### Macrolide resistance

**Fig 4:**
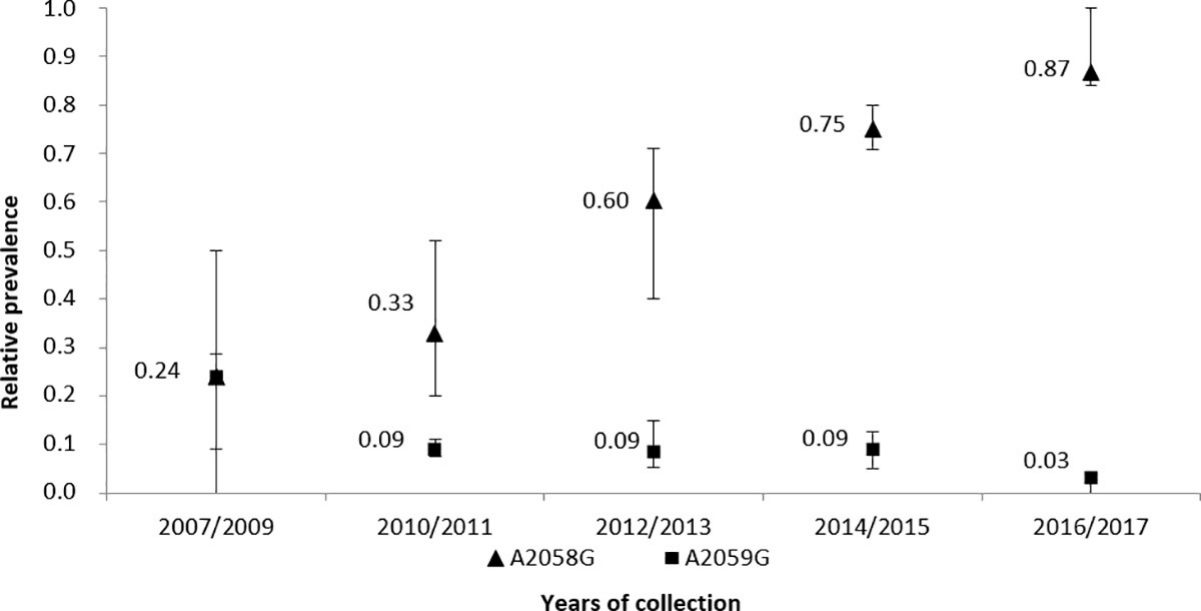
Identified prevalence of macrolide resistance causing mutations in the Czech Republic during the study period. The prevalence over the two- or three-year intervals was calculated as an average; standard errors of the mean are shown. During years 2004 to 2006, none of the mutations were found.

The prevalence of macrolide resistance causing mutations among TPA isolates in the Czech Republic is shown in Fig 4. While there is an increasing trend in the number of samples containing A2058G mutations (86.7% in 2016/2017), the number of A2059G mutations has decreased over time (3.3% in 2016/2017). This trend was detected in a previous study [4]. In addition, associations of these mutations with different allelic profiles were identified. Allelic profiles 1.26.1. and 1.3.1. were found to be associated with the A2058G mutation (*p* = 0.0342 and *p* < 0.0001, respectively) and allelic profile 1.1.3. was associated with the A2059G mutation (*p* < 0.0001). Moreover, allelic profile 1.1.8. was found to be associated with macrolide susceptibility (*p* < 0.0001).

### Associations of allelic profiles with patient characteristics

Besides macrolide resistance or susceptibility, we tested possible associations of allelic profiles with sex, locality, stage of disease and serology. No allelic profile associations with serology (RPR titer) and stage of syphilis were found. Allelic profile 1.1.8. was found more frequently among women than men (p = 0.0143). When compared to Prague, allelic profile 1.26.1. showed a geographical association (p < 0.0001) with the city of Brno.

## Discussion

In this study, we examined 675 samples collected from patients suspected of having syphilis between 2004 to 2017 from four clinics, two in Brno and two in Prague. Almost one-half of the samples were PCR-positive and typeable (n = 281, 41.6%). In comparison with other MLST studies [15–17], this study represents largest collection of typed samples. Moreover, a long time period of collecting samples allowed to discover over two dozens of allelic profiles including highly diverse allele in the TP0136 locus.

The majority of the typed samples were swabs from primary or secondary syphilis lesions (82.2%), and the rest were from whole blood or tissue samples (16.7% and 1.1%). While analysis of swab samples resulted in a similar number of fully typed (i.e., samples typed at all three TP0136, TP0548 and TP0705 loci) (n = 128, 55.4%) and partially typed samples (sequenced at least one typing locus) (n = 103, 44.6%); whole blood samples analysis revealed a minority of fully typed samples (n = 6, 12.8%) and a majority of partially typed samples (n = 41, 87.2%). For swab samples, patients that had positive RPR titer were more likely PCR positive compared to RPR-negative patients (*p* = 0.0001) suggesting that the group of patients that contains both RPR-negative and PCR-negative patients is likely to include patients not having syphilis. Altogether, these findings indicate that the swab samples are more suitable for molecular typing of TPA strains and isolates, an observation that was noticed in previous studies [4–5, 27–28].

**Fig 5:**
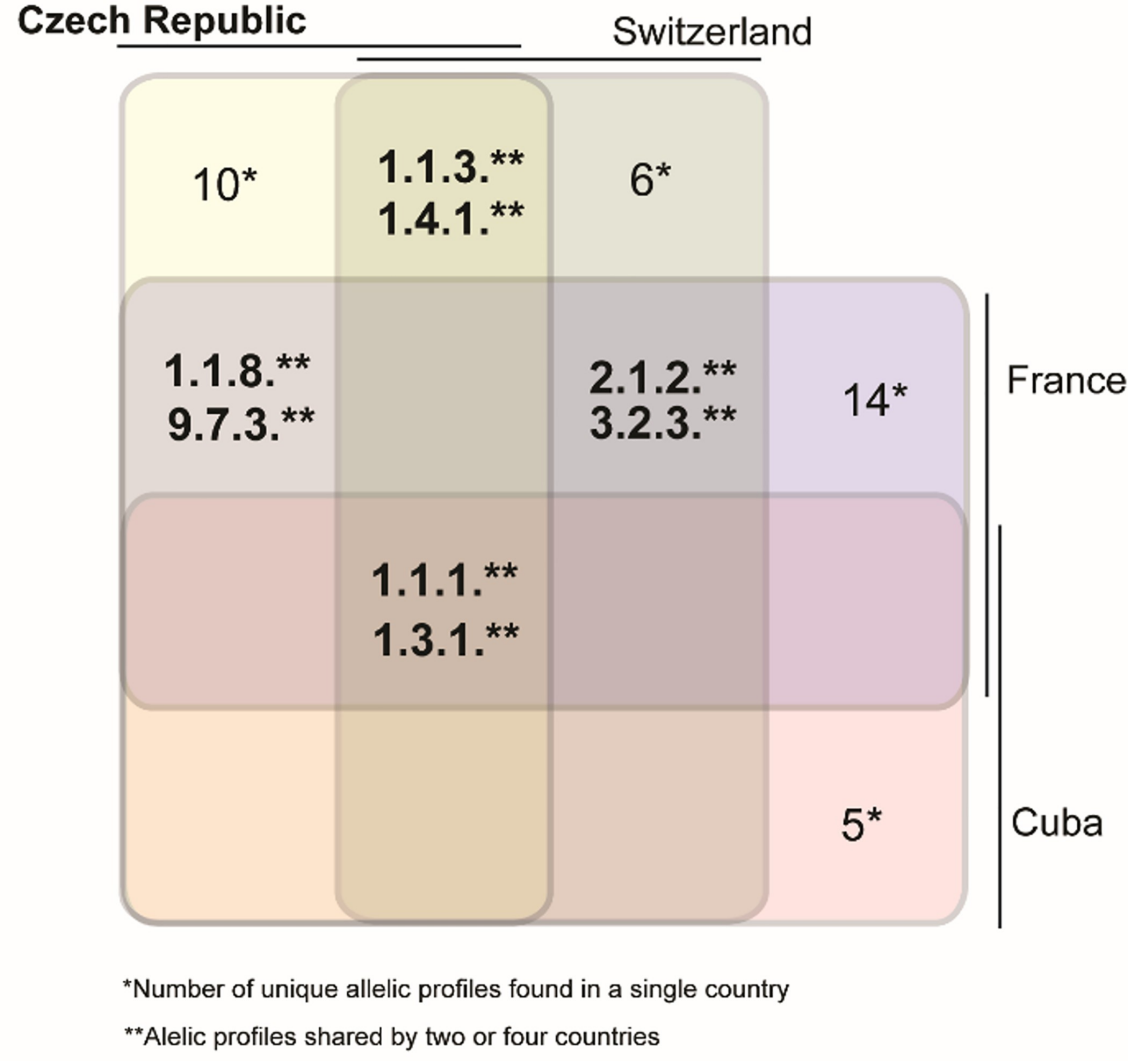
TPA allelic profiles identified in the Czech Republic, France, Switzerland and Cuba [15–18].

In this study, we have identified 16 different allelic profiles among 136 fully typed TPA-containing samples, in the same collection of samples we would identify 13 different profiles by SBMT. In the Czech Republic, in comparison to samples analyzed in previous studies from other countries [15–17], there were partial overlaps with identified fully determined allelic profiles. Samples collected in Switzerland [18] revealed four allelic profiles also found in this study (i.e., 1.1.1., 1.1.3., 1.3.1. and 1.4.1.), while 20 were different. Similarly, samples collected in France [18] revealed four shared allelic profiles (1.1.1., 1.3.1., 1.1.8., 9.7.3.) with 28 profiles that were different. Cuban samples [18] revealed two shared allelic profiles (1.1.1., 1.3.1.) and 17 different profiles (Fig 5). Only allelic profiles 1.1.1. and 1.1.3. were shared by all four countries indicating that TPA allelic profile variability is relatively high even in somewhat closely related geographical regions (Fig 5). Comparison of shared allelic profiles among Czech Republic, France, Switzerland and Cuba (Fig 5) revealed that the profiles 1.1.1. and 1.3.1. were detected in every MLST study in most time points whereas the profile 1.4.1. was detected in Switzerland at least two years earlier than in the Czech Republic (see PubMLST database [18]). Allelic profile 9.7.3. shared between Czech Republic and France was detected in similar years and profile 1.1.8. disappeared in 2016 in France and in 2016/2017 in the Czech Republic. The most frequent allelic profiles were thus similarly detected in different countries suggesting supranational spreading of certain syphilis strains. When both partially and fully typed samples were analyzed, a total of eight new alleles were identified in 281 typeable samples. We found five novel allelic variants among fully typed samples and three novel allelic variants among partially typed samples. This finding further shows the relatively high variability of TPA strains circulating in the Czech Republic.

The most common allelic profile in the Czech Republic, 1.3.1. (41.2%), corresponds to the SU2 genotype based on SBMT [19] and to the “g” ECDCT_TP0548 subtype based on ECDCT [13]. This allelic profile is also the most common in Belgium [27], France [16], Switzerland [15], Italy [29], the UK [30], and other countries including Cuba [17], the USA [31], and Australia [32]. The second and third most common allelic profiles found in this study were 1.1.8. (20.6%) and 1.1.1. (14.7%) both having allelic variant TP_0548_1 corresponding to the “f” ECDCT_TP0548 subtype [13], which is the most common in Argentina [33], China [34–39], Taiwan [40], and Russia [41].

In our study, we found differences in the local distribution of allelic profiles between samples from Brno and samples from Prague. In Prague, we found a greater number of allelic profiles, which may be related to Prague’s larger population as well as the larger number of people visiting Prague, who could represent potential carriers of new profiles. Despite the lower number of different allelic profiles identified in Brno, one profile (1.26.1.) was exclusively present in the Brno region (found in 12 patients). This finding further extends previous findings that showed differences between individual countries and suggests that sexual networks can differ even within a single country.

In this study, associations of different allelic profiles 1.26.1., 1.3.1., and 1.1.3. with macrolide resistance and 1.1.8. with macrolide susceptibility, were found. Similar associations have been found in other studies [4, 15, 42]. This study confirmed two trends: {i} there are increasing numbers of A2058G mutations and {ii} there are decreasing numbers of A2059G mutations, a trend that was also seen in a previous study [4]. As suggested by Grillová *et al*. (2014) [4], the differences in the occurrence of the A2058G and A2059G mutations could reflect opposite trends in the use of spiramycin and azithromycin in the Czech Republic. While there is a decreasing trend in the use of spiramycin, azithromycin is being prescribed more frequently [4]. While the A2058G mutation does not encode resistance to spiramycin, the A2059G does. In addition to decreased use of spiramycin, the A2059G mutation was predicted to have a higher fitness cost compared to A2058G [42].

**Fig 6:**
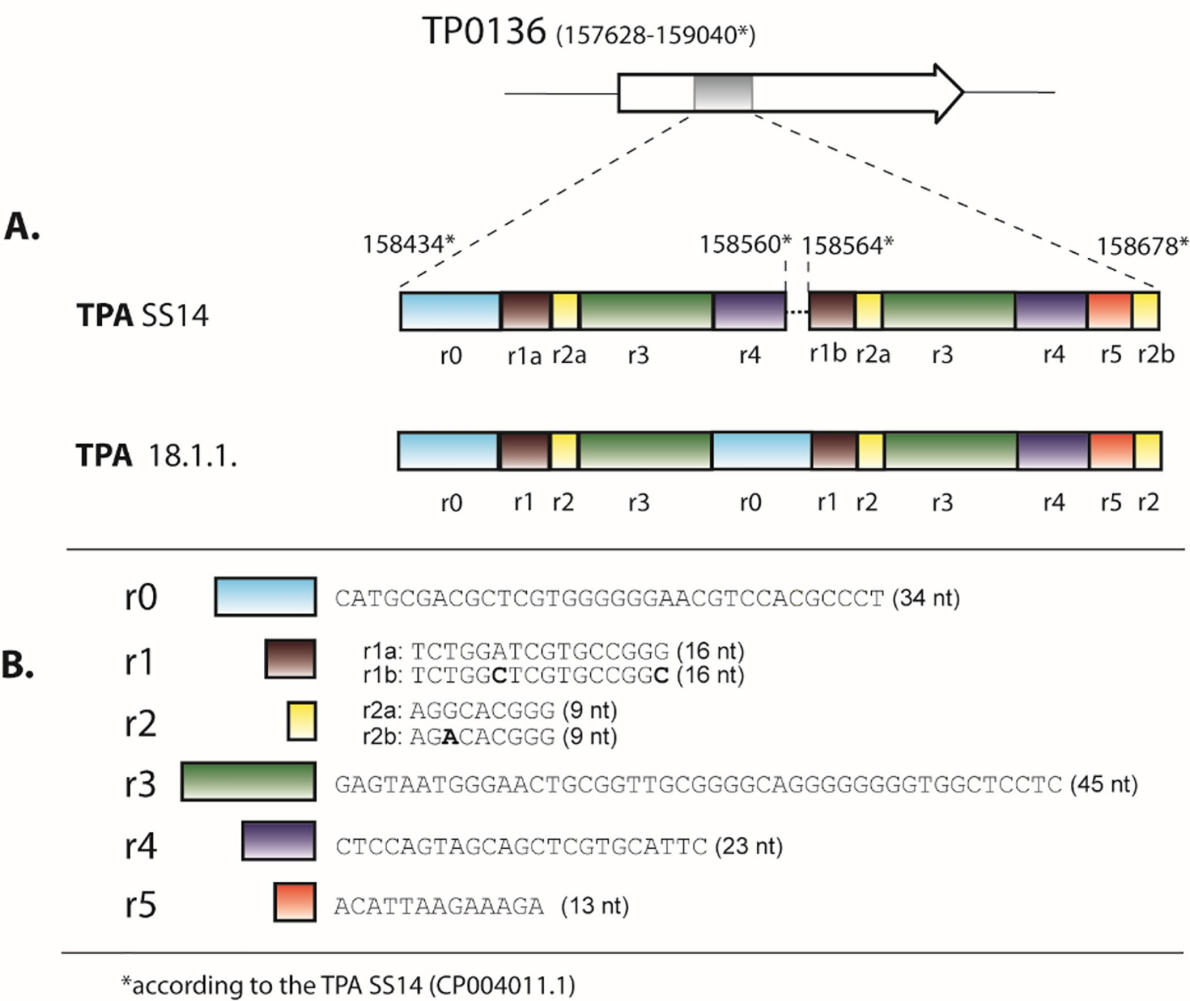
Modular structure of the TP0136 gene in the TPA SS14 strain and in the TPA 18.1.1. clinical isolate. A. A schematic representation of the TP0136 gene in the TPA SS14 strain between coordinates 158434–158678 (CP004011.1). The figure was modified from Strouhal *et al*. (2018) [45]. The r0 repetitive sequence in the TPA 18.1.1. clinical isolate replaced the first r4 repetitive sequence. B. A list of repetitive (r0, r1, r2, r3, r4, r6) and non-repetitive sequences (r5). The nucleotide differences within r1 and r2 repetitions in the TPA SS14 strain are shown in bold.

A phylogenetic analysis of allelic profiles found in this study clearly differentiated profiles belonging to SS14-like and Nichols-like strains (Fig 2), however, differences within the SS14 strains were supported with low bootstrap values (lower than 70%). With the assumption that the MLST typing system has about 30% of the discriminatory power of whole-genome analyses [15] and the low support for clustering of SS14-like strains, the identified SS14 strains were highly clonal showing low genetic diversity, which is also likely at the whole genome level. In fact, previous whole-genome studies that analyzed SS14-like strains found only limited genetic diversity among SS14-like strains [43–44]. Among the SS14-like allelic profiles identified in this study, allelic profile 18.1.1. (originally found by Flasarová *et al*. 2006) [14] appeared to be the most divergent (Fig 2), which was predominantly a result of a highly divergent sequence found at the TP0136 locus. In addition, the sequences of allelic profile 18.1.1. in TP0548 and TP0705 were identical to and different from the SS14 sequences, respectively. A detailed analysis of locus TP0136 revealed that the observed genetic diversity was a result of gene reshuffling of modular gene segments (Fig 6). A similar modular structure to that of TP0136 was found among TPE strains [45]. Another similar genetic rearrangement of TP0136 was found in the study of Grillová *et al*. [17], where four modular regions (r1-r2-r3-r4) were deleted.

The most probable explanation for the sequence differences in the TP0136 gene in clinical isolate 18.1.1 is a gene conversion event copying the r0 sequence instead of the first r4 sequence. This scenario explains why both r0 sequences were identical in the 18.1.1. isolate. There are already numerous examples of genome rearrangements in treponemal strains and isolates including the *tpr*K gene [46], *tpr*CDIGJK genes [47], the TP0133 gene [45, 48–50], rRNA (*rrn*) operons [51], TP0856 and TP0858, and other genes [45].

Most of the TPA samples analyzed in this study belonged to the SS14-clade [23, 52], and only 2.41% of the 249 determined samples belonged to the Nichols-like clade. The number of identified Nichols-like TPA strains is lower compared to previous studies [15–16]; however, it is not far from the worldwide estimated number of Nichols-like strains (i.e., 5.9%) [50].

In addition to geographical variability found among TPA samples taken from different countries, an analysis of the temporal occurrence of TPA allelic profiles (Fig 3) in the Czech Republic between 2004–2017 revealed an increasing number of identified allelic profiles and also differences in the spectra of identified allelic profiles over the years. While the first finding corresponds to the increasing number of collected samples during recent years, the second observation is consistent with the dynamic character of TPA strains in the infected population. While some allelic profiles remained for the whole study period (e.g., allelic profile 1.1.1.), other allelic profiles showed an increasing prevalence (e.g., 1.3.1.), while others slowly disappear (e.g., 1.1.8.) and some emerged (e.g., allelic profile 1.26.1. which appeared for the first time in 2012/2013 and persisted until 2017). While these findings are at least partly attributable to the random distribution of allelic profiles and to sampling bias, they could also point to possible differences in the fitness and/or pathogenicity of particular allelic profiles. As of now, we are still some distance from a determination of the full genetic diversity of TPA isolates in the global population and also from understanding the role of genetic differences in syphilis epidemiology. Mapping the genetic diversity of TPA strains in the context of additional clinical data will likely help answer at least some of these questions including connection of diverse genetic profiles with several patients´ characteristics.

## Supporting information

S1 Fig Alignment of the different allelic variants in the TP0136, TP0548 and TP0705 loci identified in this study (new alleles are in italics). (PDF)

S1 Table Primers used for the nested-PCR. (PDF)

S2 Table Allelic profiles identified in partially typed samples. (PDF)
