# Supplementary material for: MLST typing of Treponema pallidum subsp. pallidum in the Czech Republic during 2004-2017: Clinical isolates belonged to 25 allelic profiles and harbored 8 novel allelic variants
Source: PLoS One. 2019 May 31;14(5):e0217611. doi: 10.1371/journal.pone.0217611 (PMC6544256; doi:10.1371/journal.pone.0217611)
Supplement: S1 Fig — (PDF) [file pone.0217611.s001.pdf]

S1 Fig. Alignment of the different allelic variants in the TP0136, TP0548 and TP0705 loci identified in this study (new alleles are in italics).

TP0136

A. SS14-like isolates

A.Coordinatesaccordingto TPA SS14 (CP004011.1)	545-546	546-547	547-548	548-549	550	550-551	553	555	556	558	559	559-560	563	566	567	569	571	577	587	686
TPA SS14	-	-	-	-	A	-	A	C	A	C	T	-	G	T	T	A	A	C	C	G
TP_0136_1	-	-	-	-	A	-	A	C	A	C	T	-	G	T	T	A	A	C	C	G
TP_0136_4	-	-	-	-	A	-	A	C	A	C	T	-	G	T	T	A	A	T	C	G
TP_0136_17	-	-	-	-	A	-	A	C	A	C	T	-	G	T	T	A	A	C	C	A
TP_0136_18	T	A	G	GA	G	CTC	G	G	G	G	A	A	C	C	G	C	T	A	G	G

B. Nichols-like isolates

B. Coordinates according to TPA Nichols (CP004010.2)	1205
TPA Nichols	G
TP_0136_9	A

TP0548

A. SS14-like isolates

A.Coordinatesaccordingto TPA SS14 (CP004011.1)	154	155	158	163	167	448	485	488	491	503	516	772	773	775	965
TPA_SS14	G	G	G	A	G	T	C	G	G	A	G	A	A	G	A
TP_0548_1	G	G	G	A	G	T	C	G	G	A	G	A	A	G	A
TP_0548_3	A	G	A	A	G	T	C	G	G	A	G	A	A	G	A
TP_0548_4	A	G	A	A	G	T	C	A	G	A	G	A	A	G	A
TP_0548_5	A	G	A	G	G	T	C	G	G	A	G	A	A	G	A
TP_0548_26	A	G	A	A	G	T	C	G	G	A	G	G	A	G	A
TP_0548_27	A	G	A	A	G	T	C	G	G	A	G	G	A	G	G
TP_0548_28	A	G	A	A	G	T	C	G	G	G	G	A	G	G	A
TP_0548_29	A	G	A	A	A	T	C	G	G	A	G	A	A	G	A
TP_0548_30	A	G	A	A	G	T	C	G	G	A	G	A	A	G	A
TP_0548_31	A	G	A	A	G	T	C	G	G	A	G	A	A	T	A
TP_0548_32	G	G	A	A	G	T	C	G	G	A	G	A	A	G	A
TP_0548_33	G	G	G	A	G	C	C	G	G	A	G	A	A	G	A
TP_0548_34	G	G	G	A	G	T	C	G	G	A	T	A	A	G	A
TP_0548_35	G	G	G	A	G	T	A	G	A	A	G	A	A	G	A
TP_0548_36	G	A	G	A	G	T	C	G	G	A	G	A	A	G	A

B. Nichols-like isolates

B. Coordinates according to TPA Nichols (CP004010.2)	170	208
TPA_Nichols	G	C
TP_0548_7	A	A

TP0705

Coordinatesaccordingto TPA SS14 (CP004011.1)	1498	1516	1517	1828	1844	1873	1948	2111	2122
TPA SS14 and TPA Nichols	C	G	C	G	C	A	C	C	G
TP_0705_1	C	G	C	G	C	G	C	C	A
TP_0705_3	C	A	C	G	C	A	C	C	G
TP_0705_8	C	G	T	G	C	A	C	C	G
TP_0705_9	C	G	C	G	C	A	C	C	A
TP_0705_10	C	G	C	G	C	A	C	C	G
TP_0705_14	T	G	C	G	C	A	C	C	G
TP_0705_15	C	G	C	A	C	A	C	A	G
TP_0705_16	C	G	C	G	A	A	A	C	G
