# Supplementary material for: MLST typing of Treponema pallidum subsp. pallidum in the Czech Republic during 2004-2017: Clinical isolates belonged to 25 allelic profiles and harbored 8 novel allelic variants
Source: PLoS One. 2019 May 31;14(5):e0217611. doi: 10.1371/journal.pone.0217611 (PMC6544256; doi:10.1371/journal.pone.0217611)
Supplement: S1 Table — (PDF) [file pone.0217611.s002.pdf]

S1 Table. Primers used for the nested-PCR

Locus	External primers (5-3)	Coordinates[1]	Length of PCR product	Internal primers (5-3)	Coordinates[1]	Length of PCR product
TP0136	AACCCGTTAGCGCCCAACAT	157804-157823	1789 bp	AGTGTCTTCCTCGTCCGTTC	158206-158225	1206 bp
	TCCCAGCTCAGCCGAATCTC	159570-159589		CACGTGGTGGTGTCAAACTT	159392-159411	
TP0548	TGGGGCACTAAACCGGAAGA	593136-593155	1567 bp	GCGGTCCCTATGATATCGTGT	593285-593305	1065 bp
	TACGGGCATTTGCGGATAGG	594683-594702		GAGCCACTTCAGCCCTACTG	594330-594349	
TP0705	GGTCTATATGCAGCCCTTCTTC	772663-772684	1181 bp	TGCGGCTTATCCTGATGAATAG	772917-772938	803 bp
	GCTTGAGAACGATACCGGATAC	773822-773843		TATTCTGCGGCGTTGGATAG	773700-773719	
23S rDNA[2]	CGAAGGGAAGCAGGTGTAGT	234704-234723, 283149-283168	1666 and 1658 bp	GTACCGCAAACCGACACAG	234768-234786	629 bp
	GCGCGAACACCTCTTTTTAC	236350-236369		AGTCAAACCGCCCACCTAC	235378-235396	
	GAACCGTCCCTGAAAACTCA	284787-284806				

[1]According to the Nichols genome (CP004010.2).

[2]Both copies of 23S rDNA gene were amplified
