# Supplementary material for: MLST typing of Treponema pallidum subsp. pallidum in the Czech Republic during 2004-2017: Clinical isolates belonged to 25 allelic profiles and harbored 8 novel allelic variants
Source: PLoS One. 2019 May 31;14(5):e0217611. doi: 10.1371/journal.pone.0217611 (PMC6544256; doi:10.1371/journal.pone.0217611)
Supplement: S2 Table — (PDF) [file pone.0217611.s003.pdf]

S2 Table. Allelic profiles identified in partially typed samples.

	Allelic profile[1, 4]	23S rDNA[2]	No. of samples	Genetic group[3]
**1.**	1.X.1.	R8(13)/S(3)/X(4)	20	SS14-like
	1.3.X.	R8(9)/S(1)/X(1)	11	SS14-like
	X.3.1.	R8(6)/X(1)	7	SS14-like
	X.X.1.	R8(8)/S(2)/X(7)	17	NA
	X.3.X.	R8(3)/X(3)	6	SS14-like
	1.X.X.	R9(1)/S(4)/X(6)	11	SS14-like
**2.**	1.1.X.	R9(1)/S(9)	10	SS14-like
	X.1.X.	R8(1)/R9(3)/S(3)/X(2)	9	SS14-like
**3.**	X.1.8.	S(6)/X(1)	7	SS14-like
	1.X.8.	R8(1)/S(1)	2	SS14-like
	X.X.8.	R8(1)/S(1)/X(2)	4	NA
**4.**	1.X.3.	R9(5)/X(1)	6	SS14-like
	X.1.3.	R9(1)	1	SS14-like
	X.X.3.	R8(1)/R9(1)/X(1)	3	NA
**5.**	X.26.1.	R8(3)/X(2)	5	SS14-like
	1.26.X.	R8(2)	2	SS14-like
	x.26.X.	R8(1)	1	SS14-like
**6.**	1.36.X.	S(2)	2	SS14-like
**7.**	X.7.3.	R8(2)	2	Nichols-like
	9.X.3.	R8(1)	1	Nichols-like
	9.X.X.	X(1)	1	Nichols-like
**8.**	1.30.X.	S(1)	1	SS14-like
**9.**	1.27.X.[5]	R8(1)	1	SS14-like
**10.**	X.1.10.	X(1)	1	SS14-like
	X.X.10.	X(5)	5	NA
**11.**	X.35.1.	R8(1)	1	SS14-like
**12.**	X.33.3.	R9(1)	1	SS14-like
**13.**	X.28.1.[5]	X(1)	1	SS14-like
**14.**	X.32.10.[5]	X(1)	1	SS14-like
**15.**	X.34.X.	R9(1)	1	SS14-like
**16.**	X.X.14.[5]	R8(1)	1	NA
**17.**	X.X.15.[5]	R8(1)	1	NA
**18.**	X.X.9.	R8(1)	1	NA

[1] Allelic profiles based on sequences of TP0136, TP0548, and TP0705 [15].

[2] Locus encoding resistance to macrolide antibiotics: S=sensitive, R8=A2058G mutation, R9=A2059G mutation, X=undetermined. Both A2058G and A2059G mutations result in resistance to macrolide antibiotics.

[3] According to Nechvátal *et al.*, 2014 [23].

[4] Possible same allelic profiles are grouped together

[5]Newly identified alellic profiles.
